# Recent Progress in Lab-On-a-Chip Systems for the Monitoring of Metabolites for Mammalian and Microbial Cell Research

**DOI:** 10.3390/s19225027

**Published:** 2019-11-18

**Authors:** Esma Dervisevic, Kellie L. Tuck, Nicolas H. Voelcker, Victor J. Cadarso

**Affiliations:** 1 Department of Mechanical and Aerospace Engineering, Monash University, Clayton, VIC 3800, Australia; esma.Dervisevic@monash.edu; 2 School of Chemistry, Monash University, Clayton, VIC 3800, Australia; kellie.tuck@monash.edu; 3 Monash Institute of Pharmaceutical Sciences (MIPS), Monash University, 381 Royal Parade, Parkville, VIC 3052, Australia; Nicolas.Voelcker@monash.edu; 4 Commonwealth Scientific and Industrial Research Organization (CSIRO), Clayton, VIC 3168, Australia; 5 The Melbourne Centre for Nanofabrication, Australian National Fabrication Facility-Victorian Node, Clayton, VIC 3800, Australia; 6 Department of Materials Science and Engineering, Monash University, Clayton, VIC 3800, Australia

**Keywords:** microfluidic devices, optical and electrochemical metabolite sensing, intracellular metabolites, extracellular metabolites, cell culture, continuous monitoring, bioprocess monitoring, high throughput analysis

## Abstract

Lab-on-a-chip sensing technologies have changed how cell biology research is conducted. This review summarises the progress in the lab-on-a-chip devices implemented for the detection of cellular metabolites. The review is divided into two subsections according to the methods used for the metabolite detection. Each section includes a table which summarises the relevant literature and also elaborates the advantages of, and the challenges faced with that particular method. The review continues with a section discussing the achievements attained due to using lab-on-a-chip devices within the specific context. Finally, a concluding section summarises what is to be resolved and discusses the future perspectives.

## 1. Introduction

Cells are the building blocks of living organisms. They are essential in many research areas including cell biology, tissue engineering, pharmacology, toxicology, and many others [1,2,3]. The importance of research involving cells cultured in different formats including 2D and 3D bring about the necessity of developing appropriate cell culture monitoring systems. When assessing the instant status of a cell culture/organoid, a monitoring system may target extracellular or intracellular (bio)molecules, physical parameters such as pH or oxygen, or cells directly to analyse their shape, proliferation, etc. [4]. Such systems have benefited from microfluidics in terms of providing miniature, non-invasive, and high-throughput devices giving the ability to control and monitor the extracellular microenvironment on a real-time basis with reduced costs [5].

Cellular vitality depends on numerous biochemical reactions, which are part of the metabolism. Metabolism includes two reaction classes: (1) catabolic, in which large molecules are broken down to produce energy, usually in the form of adenosine triphosphate (ATP), and (2) anabolic, in which smaller molecules are used to form larger one, usually requiring energy [6]. A recent review covered methods using microfluidics for long-term and continuous monitoring of metabolism [7]. Here we focus specifically and detailed on lab-on-a-chip (LOC) technologies used for the detection and monitoring of metabolites, defined as the intermediate and end products of metabolism which can be short-chain fatty acids and carbohydrates, amino acids, steroids, polypeptides, vitamins, metal ions, etc. [8]. Conventional methods used for detecting and/or separating cell metabolites include nuclear magnetic resonance (NMR) spectroscopy, mass spectrometry (MS), gas chromatography (GC), and high performance liquid chromatography (HPLC) [9]. In addition, there are commercially available colourimetric and fluorimetric assays usable with a UV-vis spectrophotometer or fluorescence microscope/spectrophotometer, respectively. These methods are well established, having high sensitivity and reproducibility, and are capable of separating and structurally identifying the target molecule. On the other hand, these methods rely on expensive instruments and are often very time, labour, and economic resources intensive, and are mostly not applicable to the continuous monitoring systems. Those analytical methods are extensively reviewed elsewhere [10,11,12], and will not be further discussed here.

LOC refers to a miniaturised sample manipulation platform capable of performing lab-scale functions. The use of microfluidics allows the handling of pico- to micro- litres of fluid volume, which decreases the required volume of sample, consumption of reagents, time of analysis, and costs with outstanding sensing performance characteristics in terms of sensitivity and detection limits [13]. The integration of multiple micro-sized components such as pump, valve, membrane, mixer, power source, transducer, lens etc., enable LOC devices to store, transport, separate/sort, dilute/concentrate, and analyse samples via an array of different techniques [14]. Different materials have been used for the fabrication of LOC devices, are mainly Si or polymers (such as polydimethylmethacrylate (PMMA), polycarbonate (PC), polystyrene (PS), polysiloxane (glass), cellulose (paper), cyclic olefin copolymer (COC), and polydimethysiloxane (PDMS) [15]). The fabrication process of these devices has been covered elsewhere [16,17,18,19], and hence will not be detailed in this review. As polymer science and micro/nanofabrication technologies have been advancing, a wide range of LOC devices have emerged for different applications including diagnostics, proteomics, metabolomics, genomics [4,14,20], (bio)analytical chemistry [21,22,23,24], organic chemistry [25,26], environmental monitoring [27,28], fuel cells [29], biodefence [30], as well as the monitoring of food safety and quality [31,32,33].

In this review, we focus on LOC systems featuring different transducers that were designed for the detection of cellular metabolites. The two major methods, optical and electrochemical, together with their advantage and disadvantages are discussed. This is the first review showing a collection of LOC devices that can detect metabolites produced by cells directly on chip. The review does not include LOC devices used for metabolite containing sample preparation before MS analysis or other detection techniques as this has been extensively reviewed elsewhere [9,34,35]. In addition, the use of metabolite monitoring LOC systems in different areas is discussed. Finally, an overview of the existing challenges and future perspectives is presented. 

## 2. Optical Methods

Optical sensors are one of the most widely used analytical tools in cell-based research as they provide low detection limits and high sensitivities. Selected LOC systems for optical metabolite detection are summarised in Table 1. A range of signal transduction can be utilised, and the sensing of analyte can be achieved through absorption, transmission, refraction, reflection, scattering, polarisation, fluorescence, or luminescence [36]. In this section, fluorescence and colourimetric-based LOC sensing systems for metabolite detection and quantification will be discussed. Here we focus on fluorescent and colourimetric methods as these are the main optical sensing approaches used for cellular metabolite detection on LOC devices. However, other methods such as Raman and Infrared (IR) spectroscopy technologies have been externally implemented for the detection of metabolites with high sensitivities and requiring low sample volumes [37,38,39].

### 2.1. Optical Detection of Intracellular Metabolites

Biomarkers are indicators of normal or pathogenic processes, or reactions to an exposure or intervention [61]. Biomarkers can be small molecules, carbohydrates, proteins, or nucleic acids, and they can remain inside the cells while some others are released to the extracellular environment. Reactive oxygen species (ROS) act as signalling molecules in various signalling pathways but at abnormal concentrations, they are associated with various pathophysiological conditions [62,63]. The monitoring and quantification of the dynamic concentrations of ROS cannot be achieved directly with spectroscopic methods, and hence alternate detection approaches are required. One of these approaches is to use small molecules that will chemically react with ROS resulting in an optical signal change that can be measured. It is essential that assay components are stable so that ROS can be identified and/or quantified using appropriate instrumentation. There are many commercially available assay kits for ROS analyses and often these are used in combination of LOC system for the monitoring of ROS. However, when addressing different biomarkers, this is often not the case and new components need to be developed to achieve detection. Liu et al. reported a 3D co-culture of human umbilical vein endothelial cells (HUVEC) and human glioblastoma (U87) cells in a microfluidic device to study the antioxidant effects of α-lipoic acid, catechin, and ascorbic acid by monitoring the intracellular ROS and glutathione levels [44]. The reason behind targeting ROS is that increased ROS promotes the tumour malignancy by triggering secretion of vascular endothelial growth factor (VEGF), which stimulates the vascularisation of the tumour. Glutathione is a ROS scavenger and therefore, on the addition of antioxidants, glutathione levels are expected to increase. The microfluidic device was designed to co-culture HUVEC and U87 cells to mimic the tumour microvascular structure. U87 cells were embedded in hydrogel whereas HUVEC cells were cultured in hydrogel lumen which resulted in formation of a fluidic channel from HUVEC cells surrounded by U87 cells. This channel was used to expose cells to the antioxidants. The effect of antioxidants on intracellular ROS and glutathione concentrations was monitored using confocal microscopy; by observing the fluorescence signal after the addition of a cell membrane permeable ROS probe dihydroethidium (DHE), and glutathione probe 2,3-naphthalenedicarboxaldehyde (NDA). However, the lack of on-chip transducers restricts the multi-functionality of the microfluidic device.

**Figure 1:**
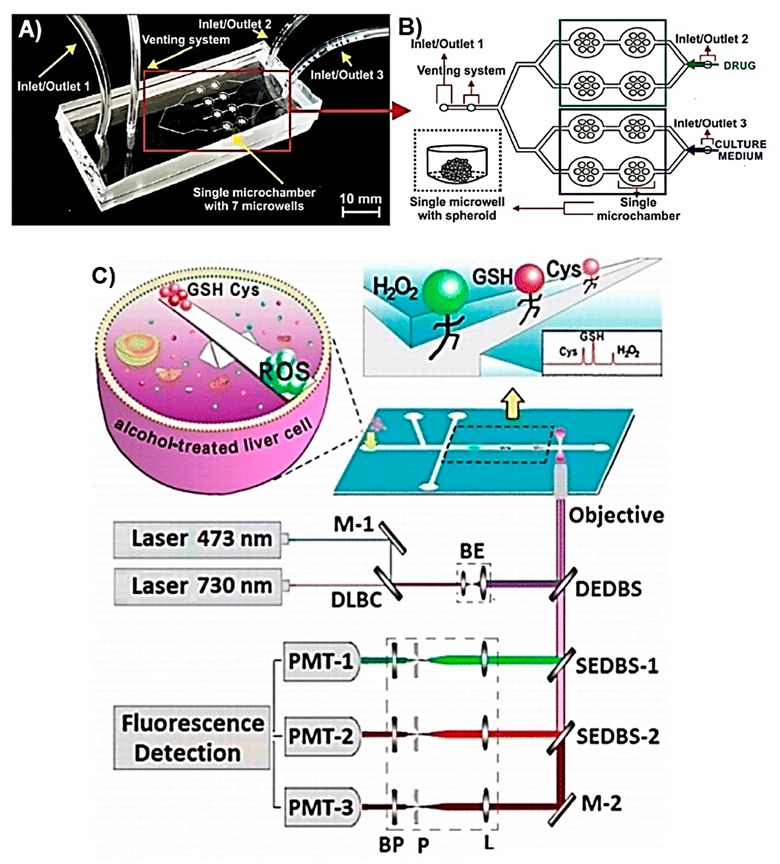
(**A**) The microsystem utilised for the assessment of PDT with the (**B**) scheme of the microsystem construction, reprinted from [48], with permission from Elsevier. (**C**) Schematic representation of multicolour fluorescence detection based microfluidic sensing system with the magnified area showing cell loading and the electrophoresis channel, reproduced with permission from [45], copyright (2016), American Chemical Society.

Intracellular ROS can also be targeted during photodynamic therapy (PDT) because the therapeutic agent usually produces high ROS concentrations in the presence of molecular O_2_ when exposed to light at a particular wavelength. Zuchowska et al. reported a microfluidic device, which is shown in Figure 1A,B, for the in vitro construction of a breast tumour model of human mammary fibroblast (HMF) and human breast adenocarcinoma (MCF-7) cells, and subsequent evaluation of PDT efficiency in the presence of *meso*-tetraphenylporphyrin (nano-TPP) [48]. The efficiency of PDT was correlated with the generation of excessive intracellular ROS and therefore oxidative stress, which as a result triggers cellular death. The practical side of this microfluidic cell culture device lies in its design, which makes it suitable for use as a conventional 384-well-plate with a benchtop spectrophotometer. The microfluidic device assembly consisted of two PDMS layers (Figure 1A). The upper layer contained the fluidic channels with the inlet and outlets, while the bottom layer contained the microchambers. The U-shaped bottom of the microchambers allows the formation of spheroids by preventing the adhesion of cultured cells. Additionally, having microchambers in the bottom layer of the device allows the exposure of spheroids to minimal flow, thus minimises shear stress. There are two inlets in the upper layer with the function of the second inlet to remove air bubbles that could damage spheroids as the culture medium is introduced into the microchambers. The exact location of nano-TPP and PDT-caused generation of intracellular ROS were both monitored using fluorescence microscopy. This study shows the successful utilisation of such a microfluidic device as an in vitro model of breast tumour spheroid corresponding to the in vivo tumour microenvironment, which consists of adjacent stromal cells, cancerous cells, and dynamic extracellular environment mimicked with microfluidic conditions such as constant flow. This LOC design allows the construction of an in vitro tumour model and subsequent analysis of intracellular oxidative stress directly on the device with either the use of a benchtop fluorimeter of fluorescence microscope. Whilst this permits the visualisation of each cell from the spheroid surface, the quantitative intracellular analysis is not possible with such a configuration. The studies mentioned above inform about the presence and concentration of metabolites from multiple cells. However, LOC systems can also allow the manipulation of a single cell in addition to the detection of intracellular metabolites using optical methods [41,45,46].

Capillary electrophoresis is a technique used to separate sample ingredients according to their charge and molecular weight in an electric field. It is a powerful analytical technique with low sample consumption, low analysis time, and high efficiency [64]. This technique has been used to separate intracellular metabolites of a single cell. Ling et al. reported a microfluidic device on which capillary electrophoresis was performed for the separation of intracellular ROS and glutathione, after which these metabolites were monitored using laser induced fluorescence (LIF) [46]. The microfluidic device was fabricated using a glass substrate and a wet chemical etch process. Human blood derived erythrocytes were initially incubated with dihydrorhodamine 123 (DHR-123), in order to fluorescently label intracellular ROS, and then cell lysis was performed on-chip by applying electrical pulse in less than 40 ms. The detection of glutathione was achieved by labelling it with fluorescent 2,3-naphthalene-dicarboxaldehyde (NDA) on the chip after cell lysis occurred, followed by analysis by fluorescence microscopy. A similar microchip electrophoresis approach was reported by Li et al., implemented for the fluorescence-based detection of intracellular ROS molecule superoxide radical (O_2_*^−^) and nitric oxide (NO), a reactive nitrogen species (RNS), from single cancerous rat adrenal gland pheochromocytoma (PC-12) cell [45]. This microfluidic device was able to transport single cells through the fluidic channel using hydrostatic pressure and electrokinetic gated injection. After the cells were incubated with cell permeating fluorescent labels that were specific for O_2_*^−^ and NO, the cell lysis, electrophoretic separation of labelled ROS and RNS molecules, and LIF monitoring were performed on the device (Figure 1C). In another study, Li et al. reported a similar microfluidic device for the simultaneous monitoring of intracellular H_2_O_2_, and the antioxidants glutathione and cysteine from single primary hepatocytes [42]. This example has advantages compared to the examples above as in addition to the function of electrophoresis, the analytes were simultaneously detected. In order to achieve simultaneous detection, the device was coupled with multiple laser light sources generating light at specific excitation wavelengths of fluorescent probes, and photomultipliers that were used to detect the emitted light coming from the sample. The advantages of microchip electrophoresis performed on microfluidic device is that targeted metabolites can be individually analysed from a single cell, and by integrating multiple fluorescence sensing systems, several metabolites can be monitored simultaneously. One of the drawbacks of this system is that due to the µs lifetime of ROS molecules, incubation of the cells with fluorescent probes prior to the on-chip analyses is needed. This can take up to 1 h and thus restricts the real-time analysis of such ROS metabolites.

### 2.2. Optical Detection of Extracellular Metabolites

#### 2.2.1. Mammalian Cells

**Figure 2:**
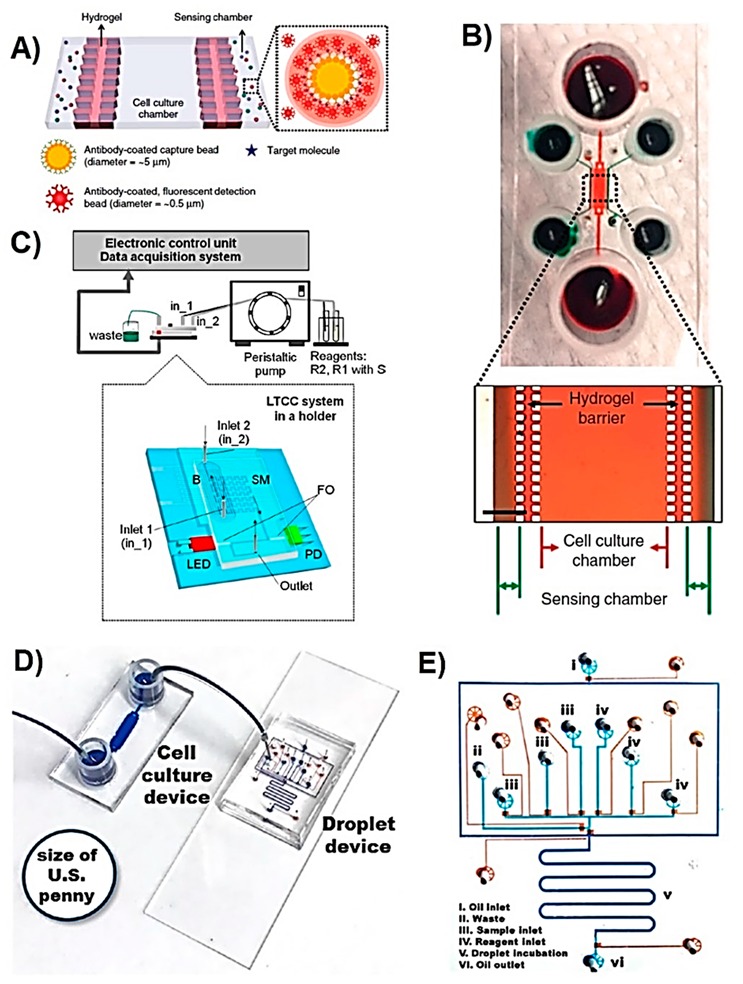
(**A**) Schematic of the microfluidic device containing a hydrogel barrier between the cell culture chamber (middle) and the sensing chambers (right-hand side). (**B**) A photograph and microscopic image of a microfluidic device containing red and green food dyes. Scale bar is 500 μm, reprinted from [55]. (**C**) Scheme of the colourimetric urea measurement system: R1 with S—the first reagent R1 containing substrate—S (urea standard), R2—the second reagent, B—microreactor with immobilised urease, SM—serpentine type mixer, FO—optic fibre, LED—light emitting diode, PD—photodetector, reprinted from [51], copyright (2017), Springer Nature. (**D**) Photographs of hepatocyte-culturing microfluidic device and droplet device. (**E**) Photograph of droplet device, reprinted from [57], with permission from Elsevier, copyright (2019).

In the case where metabolites are secreted by cells, extracellular sensing approaches need to be used. Son et al. reported a microfluidic cell culture device for the detection of hepatocyte growth factor (HGF) and the transforming growth factor (TGF)-β1 secreted by primary hepatocytes [55]. The device was fabricated using PDMS and contained three parallel channels where one, located in the middle, was used for culturing hepatocytes, and the other two, separated from the main one with a hydrogel containing PDMS pillar-like structures, contained the sensing molecules used for the fluorescent detection of growth factors (Figure 2A,B). Fluorescent polystyrene microbeads, coated with anti-HGF and anti-TGF-β1, were used to detect growth factors and increasing fluorescence was observed with increasing analyte concentration. The device was successfully used to culture hepatocytes for 7 days and detect secreted metabolites under static conditions over this period. However, this LOC system was unable to monitor metabolites in real-time due to the 45 min delay needed after the initial detectable fluorescence signal, and the requirement of changing the microbead sensor reagent after signal saturation was reached (90 min). Thus real-time monitoring still remains a challenge.

Lin et al. reported a microfluidic device for the detection of VEGF_165_ using a new iridium(III)-based luminescent complex [56]. VEGF_165_ is an angiogenic mitogen and is overexpressed in tumour cells, thus is targeted as tumour biomarker [65]. The sensing of VEGF_165_ is based on the luminescence recorded from the iridium(III)-complex which binds to the G quadruplex present in the assay mix only in the presence of VEGF_165_. The LOC device was used to mix the sample and assay reagents in a straight fluidic channel, and then the combined solution was incubated in the serpentine type channels for the fluorescence monitoring. The resulting fluorescence signal was directly proportional to the concentration of VEGF_165_. The linear range of the assay is from 0.52 to 52 pM of VEGF_156_, with a detection limit of 0.17 pM. The microfluidic device was successfully implemented to detect the VEGF_165_ from the conditioned medium of epidermoid carcinoma (CaSki) cell culture. After 3 days, 13.5 pM of VEGF_165_ was detected in the conditioned medium taken from cells grown in normal medium, and 10.7 pM in the medium containing the antimitotic drug paclitaxel, which is known to inhibit the secretion of VEGF_165_ from tumour cells. The concentrations of VEGF_165_ were confirmed using the standard method enzyme linked immunosorbent assay (ELISA). This device was successfully used for the monitoring of DNA and protein interactions, the luminescence of the resulting iridium(III)-DNA-complex can be measured within a course time of ms to min on a miniaturised analysis platform. When combined to a cell culture system, pM of metabolite concentrations could be detected in real-time thanks to the short response time of 2 s.

The urea cycle is the process that the liver performs in order to detoxify the body from ammonia. Ammonia is produced during amino acid metabolism and is excreted in the urine in the form of non-toxic urea. The urea cycle is dysregulated in cancer cells and therefore is studied in order to understand the relevant metabolism due to the underlying differences of how cancer cells use nitrogen compared to normal cells [66]. This understanding will open new targets for cancer treatment and thus among several amino acids such as arginine, glutamate, and aspartate, urea is a targeted metabolite. Colourimetric sensing is not as widely used as fluorescence-based methods because most of the commercially available metabolite sensing assays are fluorescence-based. Remiszewska et al. recently reported a microfluidic device for the colourimetric detection of urea based on a modified Berthelot‘s reaction method, which is where urea reacts with urease enzymes and then two other compounds to produce the highly coloured product indophenol [51]. In this LOC device, low temperature co-fired ceramics (LTCC) technology was used to fabricate the multi-layered device which at the end combined three major units; the serpentine type micromixer, the lamella type microreactor, and a detection cell placed between two PMMA optical fibres. One optical fibre carried light to the sample from the LED light source, and the other fibre carried the light to the photodetector coming from the sample, as shown in Figure 2C. Urease enzyme was immobilized in the microreactor and the sample analysis occurred in the detection chamber. The microfluidic urea sensing device had a linear range up to 1 mM, a detection limit of 2 µM, a sensitivity of 1.4 absorption units (AU)/µM, and a reaction time of ~10 min. The sensing system was successfully implemented for the detection of urea in conditioned cell culture media collected from hepatocyte C3A cells and from the lysate of same cells. When the samples were analysed with commercial urea assays (BioMaxima and QuantiChrom^TM^), the concentrations obtained with the sensing device were satisfactory. Although, the technology used for the fabrication of this LOC device is more complex than the one used for PDMS devices, it provides chemically and thermally stable devices. This increases the variability of the reagents to be used in the device in contrast to PDMS based devices, which are not chemically resistant to different solvents [67].

Cytokines are cell-secreted protein metabolites involved in the immune and inflammatory responses, hematopoiesis, repair and proliferation [68]. All of the aforementioned studies used adherent cells which were naturally attaching to the relevant substrates. However, suspension cells, such as lymphocytes, do not naturally attach on the surfaces and therefore require additional effort to keep the cells inside a microfluidic device. One of the approaches commonly used is to modify the inner surface of the microfluidic device with cell capturing molecules such as antibodies. This approach was implemented by Zhu et al. in the study reporting a microfluidic device for the capturing and imaging of blood T cells followed by the detection of cell secreted cytokine interleukin (IL)-2 and interferon (IFN)-γ upon stimulation with phorbol 12-myristate 13-acetate (PMA) and ionomycin [53]. A challenge regarding the long-term detection of intracellular metabolites with fluorescent cell membrane-permeable probes is that the probe might affect normal cellular functions and therefore hinder the accuracy of the obtained results. This can be overcome by the introduction of cell stimulants such as PMA that promotes the release of the metabolites into the extracellular environment. In contrast to when the cells are exposed to the stimulant only once, if same cells are to be used for several times, the dose of the stimulant need to be strictly controlled. In the study above, the cells were used only ones and therefore stimulant does not pose a concern. The microfluidic device was constructed by using a T cell and cytokine specific antibody functionalised glass slide covered by a PDMS layer with fluidic chamber used for the sample delivery and incubation. However, the poor reusability of the device due to the need to separate the PDMS layer from glass layer and long sample treatment time of ~105 min, renders its practical use.

**Figure 3:**
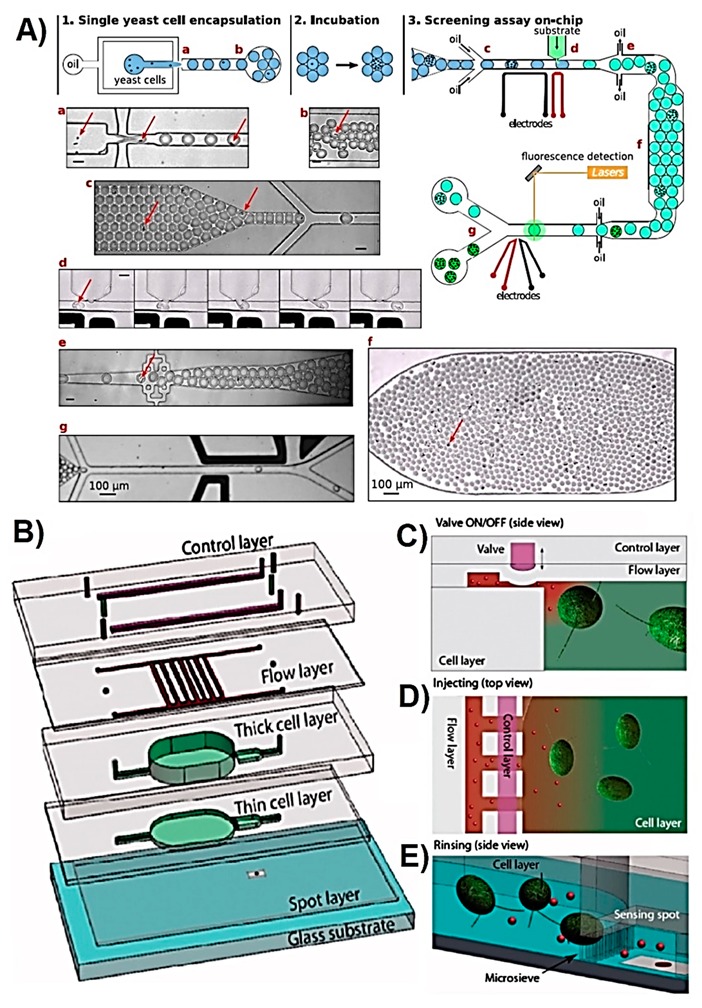
(**A**) Droplet-based microfluidic screening platform with denoted, a–f, microscopic images of the different steps of the microfluidic system. The red arrows indicate encapsulated *Y. lipolytica* cells. Unless specified, scale bars are 30 μm, reprinted from [58], copyright (2017), Springer Nature. (**B**) Multilayer microfluidic device for the optical monitoring of microalgae ROS metabolites. (**C**) Valve operation to control the connection between flow and cell layers. (**D**) Injection mode when the toxicant enters into the cell layer. (**E**) Rinsing mode when the toxicant leaves the cell layer through the microsieve, reprinted from [43], by permission of the publisher Taylor & Francis Ltd., www.tandfonline.com.

Cedilla-Alcantar et al., recently reported a LOC device hosting hepatocyte spheroid cultures, with metabolite sensing units that target lactate dehydrogenase (LDH) and bile acid 3α-hydroxysteroid, implemented for the monitoring of hepatic injury caused by palmitate (Figure 3D,E) [57]. LDH is an intracellular enzyme and can leach into the extracellular media after the cell membrane has been damaged and therefore is widely used as a marker for cellular death. Bile acids are secreted by hepatocyte spheroids and in this study were used as the marker for cellular activity. Water-in-oil droplets of 0.8 nL volume were generated in the device containing fluorescent assay components that recognise the metabolites. The microfluidic device, for both spheroid culture and metabolite analysis, was fabricated from PDMS. The culture device contained 144 microwells all connected to one inlet and one outlet. The metabolite analysis device was composed of a fluidic layer and a flow control layer used for the operation of pneumatic valves to selectively deliver metabolite specific assay reagents in the form of a drop.

The LOC device reported by Cedilla-Alcantar et al. is a great example of droplet microfluidics implemented for multiple cell secreted metabolite detection requiring ultra-low sample volume and utilising high performance liquid handling and manipulation technology. However, since the spheroid culture device has only one outlet, the analysed sample contains total metabolites secreted by all 144 spheroids and therefore acts as an analyte accumulation platform, rather than giving the ability of selectively quantifying metabolites from an individual well.

#### 2.2.2. Microbial Cells

Microbes are extensively studied due to their high importance within the clinical, environmental, and industrial context. They have been used to produce amino acids through fermentation of inexpensive carbon and nitrogen sources. Microbe-produced amino acids are used in food, cosmetic, and pharmaceutical industries and are on high demand [69]. Microbial strains are engineered to over-produce a particular metabolite that is followed by the identification of the most favourable strains. Conventional screening methods are based on accumulating the target metabolite over a certain period of time and analysing the metabolite using MS analysis or some other fluorescence-based methods. However, these methods limit the variety of detectable metabolites, require sample pre-treatment steps for intracellular metabolites and expensive equipment, and do not allow real-time monitoring. Microfluidics technology has been used to overcome these challenges. Jang et al. reported a LOC device for the high-throughput screening of L-tryptophan-producing bacteria (*Escherichia (E.) coli*) by monitoring the intracellular fluorescence from each static droplet generated on the chip [49]. The microfluidic device was fabricated using PDMS and contained a fluidic, control, and block layers. Static droplets of 100 nL volume, each containing a single bacterial cell, were generated using on-chip pneumatic valves. In order to identify the targeted bacteria, a riboswitch plasmid was used so that as the intracellular L-tryptophan concentration increases, more green fluorescent protein (sGFP) is produced and therefore, the fluorescence signal intensity increases. This LOC device enabled the analysis of 24 droplets in 20 s and is a great example of application of this technology to single cell analysis as part of library screening. In another study, reported by Beneyton et al., a droplet microfluidic device was used for the screening of recombinant enzymes produced and secreted by the yeast *Yarrowia (Y.) lipolytica* [58]. 20 pL droplets were generated in the device through hydrodynamic flow focusing containing single yeast cell and were used to grow cells for 16 h. Later on, these droplets were loaded on a second microfluidic device in which pico-injection of the reagents needed for the fluorescent assay were loaded into each droplet to analyse the activity of xylanase, cellobiohydrolase, and protease enzymes (Figure 3A). In a study reported by Abatemarco et al., droplet microfluidics was used to detect extracellular tyrosine and recombinant streptavidin produced by yeast (*Saccharomyces (S.) cerevisiae*) and perform ultrahigh-throughput microfluidic library screening [50]. The sensing technology used was named as RNA-aptamer-in-droplet (RAPID) where the target metabolite interacts with the aptamer and generates fluorescence. The droplets were also used to grow yeast cells for a few days and monitor the metabolite production, and then they were sorted according to the change in the observed fluorescence signal, thereby collecting the cells giving the greatest yield. These studies are great examples of LOC devices employed for high-throughput metabolite screening of a cell library, and are amongst many other droplet based metabolite detection platforms [70,71].

Adenosine triphosphate (ATP), the molecular unit of intracellular energy, plays important roles as a neurotransmitter [72,73,74]. Liu et al. reported a microfluidic device for the detection of bacterial intracellular ATP and ATP-conjugated submetabolites using microcapillary electrophoresis (µCE) and bioluminescence [52]. In order to detect cellular ATP, the sample of bacteria (*E. coli* strain BL21) was first introduced into the chip from the sample reservoir (SR). There, sample preparation was performed by incubating the cells with lysis buffer for 3 min. After the sample was transported into the first encountered junction on the chip, the potential applied in each reservoir facilitated the movement of ATP via reverse electroosmotic forces (EOF). In conventional µCE, EOF is used to separate molecules. However, in this study those forces were not strong enough for ATP separation. Instead, the inner walls of the PDMS fluidic channels were coated with cationic surfactant cetyltrimethylammonium chloride (CTEC) or didodecyldimethylammonium bromide (DDAB), which showed a higher efficacy, compared to CTEC and therefore facilitated reverse EOF. The reagents for bioluminescence-based ATP detection assay containing luciferase, luciferin, MgSO_4_, EDTA, dithiothreitol DTT, and bovine serum albumin (BSA) were delivered from a reservoir.

Luciferin reacted with ATP in the presence of luciferase and Mg^2+^ ions forming fluorescent product oxyluciferin. By applying different potential to each reservoir, the reaction products were transported into another reservoir during which the fluorescence of oxyluciferin was monitored. The detection of extracted cellular ATP was achieved in 30 s, with a linear concentration range of 0.2 to 50 µM, and a detection limit of 0.2 µM. The real sample analyses revealed a concentration of 1.62 amol/*E. coli* cell. In addition, this system was used to determine the formation of ATP-conjugated metabolites by monitoring the decrease in the ATP concentration. Galactose was chosen for this purpose due to its ability to react with ATP in the presence of galactokinase and Mg^2+^ ions forming 1-phosphate and ADP. The decrease in the bioluminescence of ATP indicated increased galactose concentration. The linear range for galactose was from 10 µM to 1 mM. Urine samples were analysed and the galactose concentration was found to be between 0.17 to 0.45 mM. Overall, such a device is an excellent example for a LOC system enabling the separation and detection of the intracellular metabolite from a single bacterial cell.

ROS are also targeted in order to evaluate the oxidative stress level of cells that is known to increase in the presence of nanomaterials [75,76]. The ecotoxicity of novel nanomaterials was evaluated by Koman et al. with a LOC device hosting green microalgae *Chlamydomonas reinhardtii* which were exposed to toxic CdSe/ZnS quantum dots (Qdots) and Cd^2+^ ions [43]. The cytotoxicity was assessed based on the extracellular H_2_O_2_ quantity, which is an indicator of oxidative stress. The suspension cells were cultured in the multilayered PDMS microfluidic device, which contained hydrolytic microvalves and microsieves used to separate the cells from the detection area, as illustrated in Figure 3B–E. The concentration of H_2_O_2_ was correlated with the absorbance of cytochrome C, which reacted with H_2_O_2_ changing the protein’s absorbance profile. The device allowed for continuous, non-invasive, and colourimetric toxicity monitoring with a detection limit of 40 nM of H_2_O_2_ with automated sample loading, mixing, and device rinsing features. This study investigated the total oxidative stress based on only H_2_O_2_ concentration. However, further investigation is needed to determine the selectivity of the sensor in the presence of other ROS/RNS types which potentially can also oxidise cytochrome C.

The analytical performances of the LOC systems used for the optical metabolite detection are represented in Table 1. These multifunctional platforms detected a wide range of metabolites down to pM concentrations within a single cell. The diversity of the metabolites depend on the studied cell type, which included mammalian, bacterial, yeast, and microalgae cells. The intrinsic functional components of the microfluidic devices enabled multitasking ranging from cell capture, individual or co-cultured cell or spheroid culture, efficient mixing and delivery of the reagents, pL droplet formation and manipulation, sample preparation and separation together with the sample analysis. The fluorescent analysis was performed using fluorescence microscopes. On the other hand, Remiszewska et al. reported a LOC platform containing inbuilt miniaturised light source and photodetector components allowing the metabolite detection without the need for a benchtop microscope [51]. The majority of the commercially available assays used to detect or quantify metabolites are fluorescence-based, therefore a simple microfluidic device that can assist on the cell culture or sample treatment is sufficient for the metabolite analysis while being used with a fluorescence microscope. Nevertheless, the use of colourimetric assays require a light microscope to visualise, and a spectrophotometer to quantify the targeted metabolite and thus require more complicated system designs. Overall, when combining biological sciences with optofluidic ones [13,77], the potential applications could be remarkably extended giving rise to portable, high-throughput, and highly sensitive miniaturised platforms capable of real-time monitoring of biological systems.

## 3. Electrochemical Methods for Extracellular Metabolites

LOC devices reviewed in this section contain electrochemical transducers that convert the information from a chemical reaction occurring at the electrode surface into a measurable electrical signal [78]. This electrical signal is proportional to the concentration of the analyte. Electrochemical sensors have been widely studied due to the ability to miniaturise the sensing platform and operate with low cost instruments. Advances in miniaturisation and the use of microfluidics have enabled the ability to sense pA current changes [79], resulting in a significant increase in sensitivity.

This section reviews LOC sensing devices for only extracellular metabolite detection. In contrast to optical methods, intracellular electrochemical metabolite detection is very challenging because of the need for physical contact between the working electrode and sample medium that contains metabolite, in this context that is intracellular matrix of a single cell. Several examples report nanoelectrodes able to penetrate cell membrane and detect intracellular ions [80], ROS/RNS [81], neurotransmitter, and nucleotides [82]. Although these sensing systems touch the limits of nanotechnology, they do not contain several micro/nano components as part of a LOC device (other than the nanoelectrodes) and therefore are not reviewed in here.

As summarised above in Table 2, electrochemical LOC systems have been used for the detection of metabolites from mammalian, yeast, and bacterial cells. While the achieved detection limits allowed for metabolite detection from a single cell [86], the micro components from the LOC devices enabled cell capture [89,92], cell culturing [84,85,87,88,91,94,95,97,100,102,105], automated sample manipulation (using microvalves and pressure controlled reconfigurable compartments to allow selective entrapment or release) [88,90,91,93], and sample filtration [106], in addition to the continuous and/or real-time monitoring of in vitro systems [86,87,96,97,98,100,101,102]. All of these LOC devices detected different extracellular metabolites, which were selected based on the analysis required, varying from toxicity and drug screening studies to single cell analysis. The detection of the analytes (shown in Table 2) was achieved using integrated electrodes modified with either oxidase enzymes, cytokine-specific antibodies, or used without any surface modification. H_2_O_2_ emerges as both the primary targeted metabolite and an intermediate molecule produced by the oxidase enzymes after reacting with their corresponding substrate (metabolite). The most common electrochemical method used for the continuous monitoring is amperometry. This is due to the ability to monitor the variations in the analyte concentration at a constant applied potential. The selectivity of the sensing system greatly depends on the applied potential and therefore requires to be meticulously selected when working with complex samples such as cell culture medium. Mediators that enhance the electron transfer efficiency between the analyte and recognition element are used in order to decrease the applied potential and thus interference [108]. SWV, DPV, and EIS were also used for the metabolite detection. Their limitation stems from the inability to continuously monitor the current or impedance changes. These techniques were chosen when developing immunosensors requiring long sample incubation times up to 1.5 h, in contrast to the amperometry with a response time of just a few s. In addition, the reusability of the working electrodes modified with biological elements such as enzymes or antibodies is not straightforward and we describe below how researchers have attempted to resolve this challenge [92,109,110,111]. Overall, in this section the electrochemical LOC sensing platforms used for the detection of extracellular metabolites are discussed over specific representative examples. The section is divided according to the cell type used for the relevant applications.

### 3.1. Mammalian Cells

Lactate is as a marker for anoxic cellular functions due to its accumulation during hypoxic conditions. The following examples represent LOC devices used to study single cells. The concentration of secreted lactate can vary according to the working volume of the sensing device which means that the linear range of a lactate sensor can determine the fluid volume used in the device. Lower volumes would lead to higher lactate concentrations where pL working volume result in mM lactate concentrations. Cai et al. reported a microfluidic system operating with pL volumes designed for the analysis of lactate secreted by single cardiac myocyte [94]. The microelectrodes were fabricated using photolithography and lift off methods. The detection of lactate was through the electrocatalytic oxidation of H_2_O_2_, which was generated from the reaction of lactate oxidase enzyme (ejected into the microchamber of the sensor) and lactate (released from myocytes), at an applied potential of 0.67 V. The volume delivered to the microchamber was 6.5 pL while the whole chamber had a geometric volume of 360 pL, with a sensor having a linear range of 65 to 266 fmol, and a response time of 10 s.

To prevent cells from using lactate, 15 µM of carbonylcyanide *p*-(trifluoromethoxy)-phenylhydrazone (FCCP), thus resulting in accumulation of intracellular lactate. Later on, in order to quantify the accumulated intracellular lactate concentrations of myocyte under normal conditions, ~80 µg/mL of saponin was added to increase the cellular membrane permeability and enable lactate release. The anoxic lactate production was found to be 5.1 mM, significantly higher than that of normoxic production at 1.6 mM. The concentration of intracellular lactate was calculated using the cellular volume of ~20 pL and the quantity of the released lactate.

In a study reported by Cheng et al., a microbiosensor was used to detect lactate secreted from single cardiac myocyte [95]. The working electrode was prepared by physical modification of lactate oxidase, and then electropolymerisation of poly(*o*-phenylenediamine) and an electron-carrying probe. Generation of H_2_O_2_ was monitored at 0.64 V and correlated with the concentration of lactate. The dynamic range of the lactate sensor was up to 101.5 µM and with a detection limit of 7.4 µM. Beating of the myocyte was achieved by applying an electrical field using two microelectrodes. Different potentials applied at various frequencies increased membrane permeability and thus triggered the release of intracellular lactate. The intracellular lactate concentration was calculated as 2 mM (according to the cellular volume of 10 pL). In addition to lactate, intracellular Ca^2+^ ions and pH of extracellular fluid were monitored using the fluorescent probes, fluo-3 and 2′,7′-bis-(2-carboxyethyl)-5-(and-6)-carboxyfluorescein (BCECF), respectively. The usage of fluorescent probes allowed the imaging of a single cell in the device.

In the aforementioned studies, lactate concentrations released by single cardiac myocyte were monitored in real-time. In contrast to cell cultures, where lactate accumulates in the media, for single cardiac myocytes, saponin and electrical stimuli were used to increase the lactate release and accumulation in the extracellular environment. These LOC devices are good examples of how changes at single cell level can be monitored upon different stimuli using electrochemical sensing. Several of other electrochemical LOC devices implemented for the detection of cell-secreted lactate were also reported [96,98,99,112,113]. Many of these electrochemical lactate biosensors are amperometric and operate at high applied potentials that vary between 0.45 V to 0.70 V and used to record the current changes caused by the electrochemical oxidation of H_2_O_2_ and thus these biosensors suffer from low selectivity. Notably, the use of mediators such as methylene green decreased the working potential to 0.0 V and the interference studies showed high selectivity in the presence of electrochemically active species such as ascorbic acid [96,113]. However, it is also important that interference studies involve other ROS and RNS, which may be present in the sensing media, due to their high chemical reactivity.

**Figure 4:**
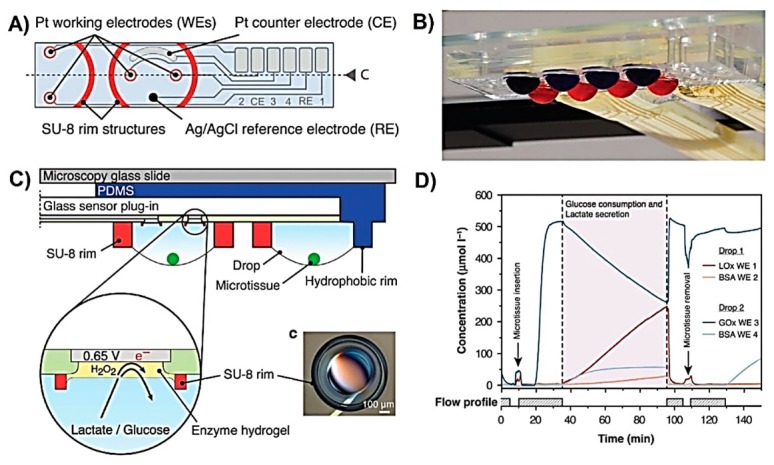
(**A**) Close-up view of the sensor unit featuring working, counter, and reference electrodes with the SU-8 rim structures indicated in red. (**B**) Photograph of an assembled device loaded with coloured liquid. (**C**) Configuration of the biosensor. (**D**) Monitoring the metabolism of HCT116 eGFP microtissues and their response to medium changes in terms of continuous measurements of glucose consumption and lactate secretion, reprinted from [100], copyright (2016) Springer Nature.

Lactate was also used as a marker for metabolic rate of a cell culture. Misun et al. reported a microfluidic device integrated with electrochemical lactate biosensor for the monitoring of human colon cancer microspheroid cultures from a hanging drop [100]. The hanging drop network was extensively characterised and optimised for the spheroid culture and analysis in previous studies [114,115]. The design for the electrochemical biosensors is shown in Figure 4A, and the assembly of whole device is shown in Figure 4C. The detection of lactate was performed through the generation of H_2_O_2_ at 0.65 V applied potential. The working electrodes were functionalised by drop-casting a mixture of lactate oxidase enzyme, BSA, glutaraldehyde, and Triton X-100 on the Pt electrodes that were coated by electrochemical polymerisation of *m*-poly(phenylenediamine) followed by vapour deposition of (3-aminopropyl)triethoxysilane. Hanging drops were obtained by patterning hydrophobic O-rings made of SU-8 enclosing an O_2_ plasma activated hydrophilic PDMS centre (Figure 4B). This allowed the fluid to be entrapped inside the SU-8 rims while positioning the microspheroids at the bottom of the hanging drop due to the gravitational force. This device was successfully used to simultaneously monitor the lactate production of human colon carcinoma (HCT116 eGFP) microspheroid cell culture from the hanging drops with a volume of 10 µL (Figure 4D). The device was used for the monitoring of multiple analytes to understand the metabolism of the microspheroids. The open microfluidic device formed of hanging drop network is favourable in terms of providing easy access to the microspheroid and the cell culture medium. On the other hand, the limitation of the present design is the relatively short lifetime of ~1 day, which restricts the continuity of the measurements. Exchanging the sensor gasket requires the transfer and re-loading of the spheroid into the device system.

**Figure 5:**
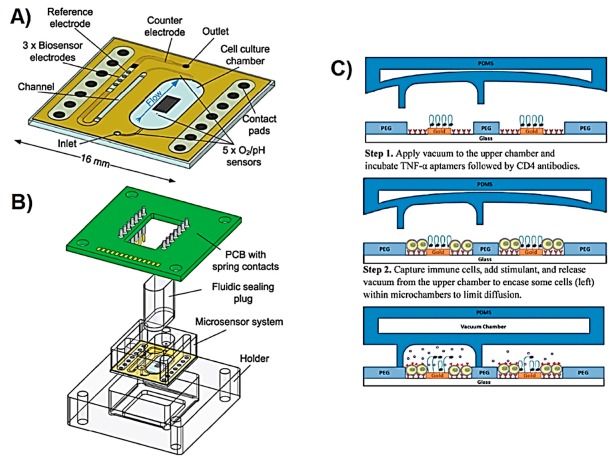
(**A**) The Illustration of the layout of the chip used for growing and monitoring human brain cancer (T98G) cells with primary features noted, (**B**) the system components and electric interface, republished with permission of Royal Society of Chemistry, from [97] (2014) permission conveyed through Copyright Clearance Center, Inc. (**C**) The illustration for the reconfigurable microfluidic device, which is actuated to lower a microcup around a selected group of cells being captured by the antibodies after which the cytokines released are detected with the biosensors, republished with permission of Royal Society of Chemistry, from [88] (2014) permission conveyed through Copyright Clearance Center, Inc.

Weltin et al. reported a LOC device for the monitoring of lactate to track the changes in the cellular metabolic rate [97]. The device was used for culturing human brain cancer (T98G) cells and then monitor lactate production upon exposure to cytochalasin B (CB), which prevents the transport of glucose into the cells. The device, illustrated in Figure 5A,B, was made of a PMMA gasket glued to a glass substrate containing sensors for lactate, glucose, pH, and O_2_. Lactate was monitored through the electrochemical oxidation of H_2_O_2_ at 0.45 V applied potential. The interference issues that could arise because of the high working potential were addressed by calibrating the sensor. The calibration was performed by measuring the current at the working potential with a bare electrode, which allowed the electrochemical oxidation/reduction of molecules from the culture medium other than lactate. The linear range of the sensor was up to 10 mM with a detection limit of 75 µM of lactate. The changes in the cell metabolism after exposure to CB were recorded in terms of decreased lactate production and decreased acidification of the cell culture medium. The latter was attributed to the reduced metabolic reactions involving the production of CO_2_ that dissolves in the medium and reduces pH.

Cytokines are peptides that facilitate the intercellular communication within the immune system. They are studied as therapeutic agents or as therapeutic targets [116]. As also seen for optical sensors, cytokines released from various cells were electrochemically detected using different LOC systems. Some of the sensing devices were used for culturing cells while some others for cell capture and cell culture due to the aforementioned challenges (in Section 2.2.1) faced with suspension cells. In some cases, cell capturing allowed the selective separation of cells from a complex sample such as blood. Liu et al. reported a LOC device for the selective capturing of T-lymphocyte cells from blood samples and monocyte (U937) cells, and detection of TNF-α and IFN-γ secreted from the captured cells upon stimulation with 50 ng/mL PMA and 2 mM ionomycin [89]. The sensing device contained two fluidic channels each having four Au microelectrodes with a diameter of 300 µm. Ag/AgCl reference and Pt counter electrodes were eternally placed in the inlet of the fluidic channel and outlet tubing of the device, respectively. The cells were captured with their corresponding antibodies immobilised around the working electrode. Electrochemical detection of proteins is more challenging because proteins are usually not electrochemically active and therefore require additional elements, such as nanomaterial or redox label, to increase the electron transfer efficiency and sensing performance characteristics. In this study, the targeted cytokines were detected using SWV recording the current changes at voltages corresponding to two redox probes: methylene blue (MB, −0.15 V) and anthraquinone (AQ, −0.37 V). MB was covalently linked to TNF-α, and AQ to IFN-γ selective aptamers, and then immobilised on the Au electrode using the thiol groups of the aptamers. As the concentration of captured cytokine increased, a decrease in the current was observed due to possible blockage of electrical conductivity caused by the proteins. This LOC device allowed simultaneous detection of cell-secreted cytokines for 2 h. However, the selectivity of the sensors needs to be explored in more details by performing interference studies due to the possibility of electrochemically reducing or oxidising other molecules at the selected potentials, such as ROS, which are also secreted by the cells upon stimulation [117]. Affinity-based biosensors are usually for single-use because the electrode surface is modified with corresponding antibody or aptamers that form irreversible chemical bonds with the targeted analyte. The surface of the electrode requires to be regenerated in order to be reusable. The reusability of the electrodes from a LOC sensing device can be advantageous in terms of increasing the lifetime of the device and thus make it more favourable for continuous monitoring. In a study reported by Zhou et al., a LOC device similar to the one explained above [89], sensing electrodes were regenerated using urea and used for the detection of the cytokine IFN-γ secreted from T cells captured by the antibodies immobilised inside the microfluidic device [92]. Regeneration is highly promising for continuous monitoring of cytokines as the biosensor demonstrated ability to operate after being regenerated for three times over 600 min.

Advanced LOC systems can enable the control over boundaries defined within the microfluidic channels. Kwa et al. reported a reconfigurable microfluidic device assisted by aptamer-based electrochemical biosensors for the monitoring of TNF-α release [88]. The device contained three layers: the first layer was a glass substrate with three aptamer-modified Au electrodes (explained in [89]). The second and third PDMS layers contained fluidic channels with pressure-controlled valves, either restricting the fluids to encounter each other or allowing them to mix (illustrated in Figure 5C). The device was used to study the intercellular communication of two neighbouring cell populations based on the released cytokine profile. In another study reported by Zhou et al. [118], a similar reconfigurable LOC device assisted by an aptamer-based electrochemical TGF-α biosensor was used to study the intercellular communication of hepatocyte and stellate cells cultured in two separate parallel fluidic channels. The design of this device allowed controlled release of cytokines to a different set of sensing electrodes by using the aforementioned valve-like system. This type of LOC device was also used to dynamically monitor the release of exosomes by hepatocellular carcinoma (HCC) cells, and INF-γ secreted by T-cells [119]. In these studies, the cells were captured with their corresponding antibodies immobilised on the bottom of the fluidic channels. This approach allowed for the selective capturing of targeted cells even from a complex sample such as blood, requiring less waiting time for the cells to attach to the surface. On the other hand, antibodies are relatively expensive and therefore increase the relevant costs. The reusability of the LOC device, including the integrated sensors, will significantly decrease the time and expenses required for fabricating the device.

**Figure 6:**
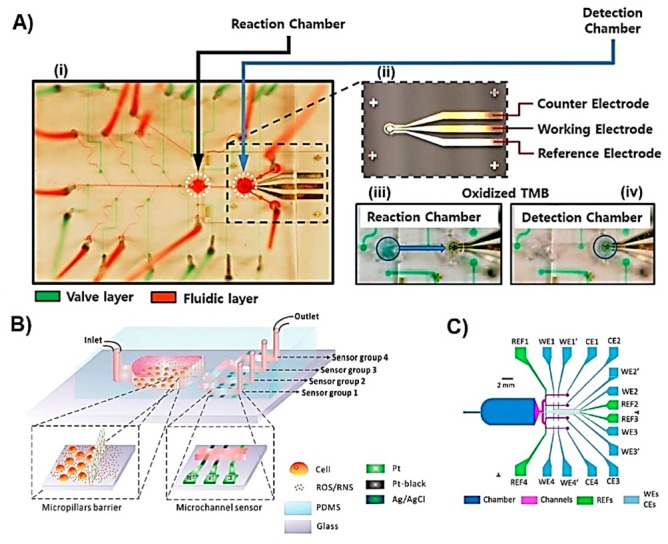
(**A**) Configuration of the fabricated microfluidic sensing chip, (i) photograph of the microfluidic chip with an integrated microelectrode, (ii) only the microelectrode, (iii) the reaction chamber with oxidised TMB, (iv) transfer of oxidised TMB to the detection chamber, reprinted from [93], Copyright (2016) Springer Nature. (**B**) 3D scheme of the microfluidic platform integrating a microchamber upstream for cell culture and four parallel channels with microband electrodes downstream. (**C**) Top view of the microdevice with its microfluidic circuit and electrode paths for electrical connection, reprinted with permission from [85], copyright (2018) American Chemical Society.

The liver is the major site for metabolism and drug biotransformation and thus many microfluidic systems used for drug toxicity have been developed using hepatocyte cells [120,121,122]. Riahi et al. reported a LOC device integrated with microvalves and a microelectrode for the continuous monitoring of hepatocyte-secreted proteins in the presence of toxic drug acetaminophen (APAP) [93]. APAP is usually used to validate the newly developed LOC systems due to its known hepatotoxicity. In this example, the comparison of cytokine release profile of healthy cells and APAP treated cells inform about the toxicity of the tested compound and thus applicability of the LOC system for the drug toxicity studies. The device was composed of two fluidic PDMS layers, one for samples and another for the pressure controlled valves, separated by a 40 µm thick PDMS membrane (Figure 6A(i) and (ii)). ELISA-based sensing approach was used to selectively detect transferrin (TF) and ALB. The substrate of conjugated HRP was 3,3′,5,5′-tetramethylbenzidine (TMB) and the concentration of the captured proteins was correlated with the current changes obtained from H_2_O_2_ generation at −0.1 V applied potential. The device contained a reaction chamber where the analytes were incubated with relevant antibodies, and a detection chamber containing the sensors (Figure 6A(iii) and (iv)). Avoiding direct surface modification of the microelectrodes allowed an improvement in the reusability and lifetime of the sensor. The linear range was 10–4000 ng/mL for TF, and 15–4000 ng/mL for ALB, both having detection limits of 0.03 ng/mL. The microfluidic device was coupled with a microbioreactor housing hepatocyte (HepG2) cells which were exposed to cytotoxic drug APAP. The TF and ALB secreted by the cells were monitored for up to five days with high reproducibility and correlation with the results obtained with commercial ELISA-based TF and ALB assays. The microfluidic device is highly versatile and reliable but the total analyte detection time of 105 min and thus demands on further improvements to enable real-time metabolite monitoring.

Zhang et al. reported a modified version of the cell culture monitoring system reported in [93], by integrating three immunosensors for the monitoring of cell-secreted ALB, glutathione S-transferase α (GST-α), and creatine kinase MB (CK-MB) coming from two microbioreactors hosting human liver and cardiac organoids [90]. The target proteins released upon activation with PMA or ionomycin, were detected using EIS in the presence of redox couple ferricyanide/ferrocyanide ([Fe(CN)_6_]^4−/3−^). In the case where the electrode surface does not contain a redox marker, a redox probe can be added to the sample medium to facilitate sufficient electron transfer efficiency. Although amperometry-based analysis techniques highly demand on a redox marker in order to decrease the working potential, EIS type of techniques require an electrically conductive sample medium rather than a redox marker. [Fe(CN)_6_]^4−/3−^ is one of the most commonly used redox probes in addition to hydroquinone, benzoquinone, and hexamineruthenium [123]. The total analysis time for the sample was 58 min with a linear range of 0.1–100 ng/mL for ALB, 0.1–100 ng/mL for and GST-α, and 0.01–10 ng/mL for CK-MB. The long-term reusability was achieved by cleaning the surface of the electrode with H_2_SO_4_ and K_3_Fe(CN)^6^ for 18 min. The cleaning was needed as the sensor reached saturation within approximately 200 min post-activation of cells. An additional optical sensing unit (a microfluidic device) was integrated for the monitoring of pH and O_2_. These two sensors allowed for assessing the functionality of the microbioreactors. The entire system reported in this work shows promise for establishing biomimicking organ-on-a-chip models for cytotoxicity analyses due to the capability of maintaining organoids and quantifying cellular metabolites for long time thanks to the integrated bubble trap, the sensors, and the microvalve systems.

ROS and RNS, such as H_2_O_2_ and NO*, can be targeted to study the oxidative stress of chemical, physical, or electrical stimuli on different cells. Matharu et al. reported a microfluidic device used to culture hepatocyte cells and to study the alcohol exposure-caused oxidative stress by monitoring the amount of the released H_2_O_2_ [84]. The sensing of H_2_O_2_ was performed by immobilising a composition of PEG and HRP enzyme on a Au working electrode that was surrounded by the cells. The exposure of cells to alcohol caused the release of H_2_O_2_. The current generated from the oxidation of HRP by H_2_O_2_ was monitored at −0.4 V applied potential. The detection limit was 0.2 µM and the linear range was up to 100 µM of H_2_O_2_. The results were compared with the use of the fluorescent 5-(and 6)-chloromethyl-2′,7′-dichlorodihydrofluorescein diacetate (CM-DCFDA) probe, which is an indicator for intracellular oxidative stress due to its ability to react with several ROS types including H_2_O_2_. The cells were studied in very close proximity to the sensing electrodes using low total sample volumes of 3 µL. The total H_2_O_2_ concentration released from ~12,000 cells was estimated to be 1.16 µM (0.29 fmol/cell with 3 µL working volume) at the end of exposing the cells to alcohol for 3 h. The ability to work with small volumes allows decreasing the number of cells, the amount of chemical and reagents required and thus lowering operational costs. Since any molecule released from cells is in very low quantity and concentration, monitoring cell-secreted metabolites using a microfluidic system is highly advantageous. However, cells can release other ROS types that might react with HRP and thus the selectivity of the sensors needs to be explored in more details. This is even more crucial in an electrochemical system such as the one proposed, where any reaction occurring at the selected potential can cause changes in the electrochemical signal, reducing specificity.

**Figure 7:**
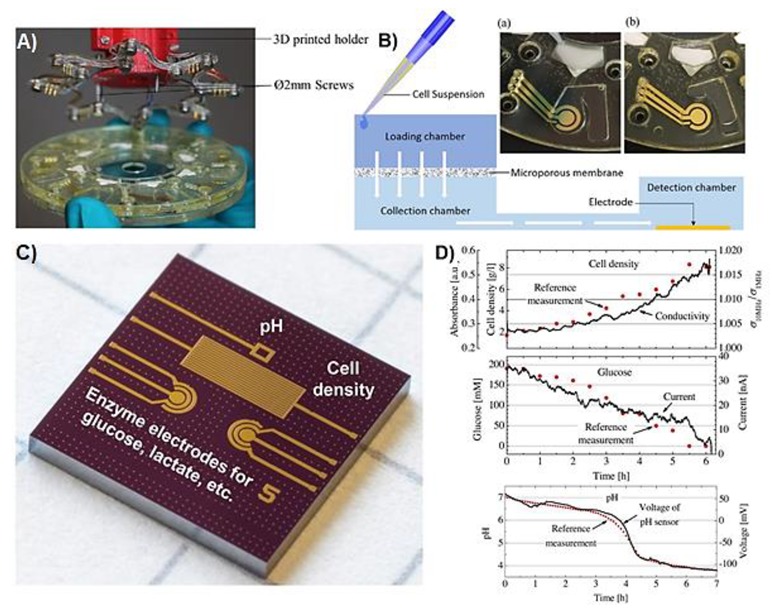
(**A**) The photograph of the microfluidic disc and printed circuit board with the magnetic clamping. (**B**) Schematics of the cross section of the microfluidic layout (the arrows show the flow direction during rotation): sample (a) before and (b) after on-disc filtration, reprinted from [106], with permission from Elsevier, copyright (2017). (**C**) The photograph of the integrated MEMS sensor chip with a size of 7.16 × 7.16 mm^2^. (**D**) Continuous bioprocess monitoring of cell density, glucose, and pH in a yeast culture, reprinted from [101], with permission from Elsevier, copyright (2016).

In a LOC system used for assessing oxidative stress, culturing cells in the device is highly advantageous because of short lifetime of ROS and RNS molecules. When the cells are close to the electrodes, the time delay between metabolite release and detection can be significantly short allowing the detection of highly unstable molecules such as peroxynitrite (ONOO^−^) that has a half lifetime of 5–20 ms in biological media [124]. It is also important that cells do not physically interfere with the sensing electrodes as that would cause damage to the electrodes and inhibit the generation of electrochemical signal. Li et al. developed a microfluidic device, as shown in Figure 7B, containing micropillars that separate and thus prevent cultured macrophage cells from penetrating into the subsequent microfluidic channels containing electrodes [85]. The device was used to study the oxidative burst of macrophage cells caused by the chemical stimulation with calcium ionophore. Simultaneous detection of extracellular H_2_O_2_, ONOO^−^, NO*, and nitrogen dioxide (NO_2_^−^) was achieved using carbonised Pt working electrodes at different applied potentials. Cumulative detection of ROS and RNS can represent total oxidative stress that cells experience however, individual quantification of each species is also of high importance because each one is involved in different downstream signalling processes [125]. The detection and quantification of each species was performed by monitoring the current changes at 0.3 V for H_2_O_2_, 0.45 V for ONOO^−^, 0.62 V for NO*, and 0.85 V for NO_2_^−^. The average quantity of each species released by a single cell was 5.1 fmol for H_2_O_2_, 6.4 fmol for ONOO^−^, 7.9 fmol for NO*, and 6.8 fmol for NO_2_^−^. The selectivity of the working electrode towards detecting each ROS and RNS type had been studied in microfluidic devices before by same research group showing the sensors high reliability [126,127,128].

LOC sensing devices are not only used for drug toxicity, metabolic rate, and oxidative stress studies but also for studying brain physiology and relevant disorders by constructing brain-on-a-chip model systems [129]. Norepinephrine is a hormone and neurotransmitter involved in many biological processes in the central and peripheral nervous systems and is targeted to gain new insights about pathophysiological conditions of these systems [130]. Ges et al. reported a LOC device for the monitoring of norepinephrine released from adrenal chromaffin cells [103]. The sensing electrode was fabricated by depositing Ti/Pt (10/100 nm) on a glass substrate after which iridium oxide was electrochemically deposited and used to monitor the current changes. The concentration of norepinephrine was correlated with the current obtained at 0.72 V due to the electrochemical oxidation of the metabolite. The sensor had a linear range up to 400 µM and was successfully applied for the cell-secreted metabolite detection in nL sample volume. In a similar device configuration, the cumulative quantification of catecholamines including epinephrine, norepinephrine, and dopamine secreted from neuroendocrine cells was reported [104]. The device offers the ability of real-time monitoring of metabolites secreted from on-chip trapped cells. In addition, the structure of the working electrode is composed of only metallic compounds, which allows for reusability of the sensors. On the other hand, the selected potential of 0.72 V might lead to the electrochemical oxidation or reduction of other cell-secreted metabolites and thus rise concerns about the selectivity of the sensor.

### 3.2. Microbial Cells

Infection by pathogenic microbes can have detrimental effects and thus microbes are used for identifying antibiotic susceptibility and discovery of new drugs [131]. Nonetheless, non-pathogenic microbes can be used to produce valuable compounds with various industrial applications [132].

*p*-Coumaric acid (*p*HCA) is a secondary metabolite acting as an antimicrobial, antioxidant, analgesic, anti-inflammatory, and immunoregulatory agent naturally present in plants. Engineering bacteria is one of the methods used to produce large amounts of *p*HCA as an alternative to the more expensive methods including extraction from plants [133]. Sanger et al. reported a lab-on-a-disc (LOD) platform for the detection of *p*HCA produced by *E. coli* bacteria in order to quantify the metabolite from cell culture media as part of screening the modified strains [106]. The device was fabricated using four layers of PMMA bound to each other using a double-sided pressure sensitive adhesive tape. The bottom PMMA layer included the three Cr/Au (20/200 nm) electrode system for the electrochemical sensing. A microporous cellulose acetate membrane with 0.2 µm pore size was integrated in order to filter out bacterial cells before the sample analysis occurred. In addition, small magnets were added to establish a connection to the electrochemical workstation through an additional custom-made slip ring (Figure 7A). The disk was mounted on a spin-stand that enabled the rotation of the disk and therefore traveling of fluid through the fluidic channels due to the centrifugal force without need of pumps. The sample taken from bacterial culture was loaded, filtered from the cells at 700 rpm for 5 min, and then analysed for the *p*HCA concentration using SWV (Figure 7B). The interference of other media components was avoided by building the calibration plot using spiked cell media samples, which initially did not contain *p*HCA. The dynamic range of the sensor was from 0.125 mM to 3 mM. The real sample analyses showed good correlation with the results obtained with HPLC proving the promising applicability of such a LOD sensing platform for the fast detection of bacterial secondary metabolites as a cost-effective and fast tool for screening the engineered strains. LOD technology has been developed for diagnostics and many other applications and has been extensively reviewed elsewhere [134,135,136].

A different metabolite sensing approach was reported by Bellin et al. where an electrochemical imaging chip was used for culturing *Pseudomonas* (*P.*) *aeruginosa* PA14 colony biofilm for the detection and localisation of phenazine derivatives [105]. Phenazine derivatives are nitrogen containing heterocyclic redox-active compounds that have antifungal, antibacterial, antitumor properties, and can be used as electron donor or acceptor in different processes including microbial fuel cells and electrochemical biosensors [137,138]. Microbes are used to produce valuable metabolites and thus high-throughput colony screening methods are on high demand such as the one reported by Belin et al. The electrochemical imaging chip was an integrated circuit 1 × 1 cm^2^ device custom-made using a complementary metal-oxide-semiconductor (CMOS) process followed by vapour deposition and lift off processes for the definition of electrode areas. The detection of phenazine derivatives produced by the bacteria was performed using SWV in the potential range of 0.1 to 0.7 V. The colonies were grown on a track-etched membrane and then transferred onto a chip, which accommodated 1824 electrodes having a total analysis time of 5.2 min. The phenazine derivatives phenazine-1-carboxylic acid (PCA), 5-methyl-phenazine-1-carboxylic acid (5-MCA) and pyocyanin were reduced at −0.5 V, −0.2 V, and −0.37 V, respectively. The spatial distribution of the metabolites throughout the biofilm was obtained by constructing a colour gradient with respect to the detected electrochemical signal (current) and therefore the concentration of the metabolite. The current detected at a particular potential was converted into analyte concentration using a calibration plot, and then the colour gradient is formed starting with the smallest and ending with the highest detected concentration, representing the brightest and darkest colour, respectively. The fabricated device was successfully used to electrochemically image bacterial culture localising each metabolite across the biofilm and analysing the effect of one of them on the production of the other. This approach for colony analysis could significantly improve the understanding of intercellular communication and the resulting patterning of the biofilm.

Some of the biotechnological processes that involve microbial cell cultures require the quantification of high metabolite concentrations up to hundreds of mM, such as in the fermentation processes [139]. The sensors used in such bioreactors need to have appropriate linear range and long lifetime suitable for continuous monitoring. The integrated microelectromechanical systems (MEMS) device reported by Mross et al. was used for simultaneous monitoring of glucose as the cell nutrient, lactate as the cell metabolite, pH, and cell density of microbial cell cultures (the device is shown in Figure 7C) [101]. For the detection of lactate, the working electrode was modified by drop-casting a mixture of lactate oxidase, crosslinking reagent glutaraldehyde, and BSA. Later on, polyurethane solution containing tetrahydrofuran (THF) and dimethylformamide (DMF) solvents was deposited on the working electrode area in order to limit the diffusion of biomolecules and therefore enlarge the linear range of the sensor, in addition to prevent leaching of enzyme into the media. Amperometric signal coming from the electrochemical oxidation of H_2_O_2_ was recorded at 0.7 V and was correlated with the concentration of lactate, resulting in a linear range up to 900 mM. This sensing device with a size of 7.22 × 7.22 mm^2^ was successfully implemented for the in situ monitoring of cell nutrient, metabolite, density, and medium pH of *S. cerevisiae* and *Lactobacillus* (*L.*) *acidophilus* cultures (Figure 7D).

Overall, the integration of the electrochemical sensing with the LOC technology can provide user-friendly and versatile metabolite monitoring systems for various applications involving single cells, cell culture, organoids, and microorganisms.

## 4. Achievements

Development of microfluidics is restructuring how biology is studied and researched. Conventional in vitro tissue culture techniques lack the ability to mimic exact microenvironments and therefore the functionality that is observed in the in vivo systems. To overcome this massive disadvantage, LOC devices have been used in tissue engineering, drug development, cell manipulation, and cell analysis [14]. With the ability to obtain organ-on-a-chip by mimicking the microenvironment, tissue composition, and therefore the function of an organ, the potentiality grew up to personalised medicine and disease modelling allowing the development of individual specific therapies. Further progress of the research conducted with such LOC platforms rely on advanced sensory systems to monitor the viability and metabolic activity of the cell, tissue, and organoids maintained in the device in real-time [140]. The viability and metabolic activity can be tracked by monitoring cellular metabolites, the changes in the composition of cellular microenvironment and physical parameters, or directly analysing the cell shape and motility.

In the area of metabolomics which aims to identify and quantify small molecules from the cells, tissue, organ, and biological fluids [141], microfluidics has been already benefiting NMR, LC, and MS, the most commonly used sample analysis techniques in metabolomics [142,143,144,145]. This intersection provided faster and more efficient sample preparation processes requiring sub-nL sample volume [146].

When assessing the metabolic biotransformation of a drug or its candidate, identifying and quantifying the primary metabolites is of high importance [147]. Liver is the main site of the drug metabolism and thus its secretory factors and bile acids have been monitored using LOC systems [57,93]. However, extensive evaluations need to be performed involving several other tissue and organs in order to fully understand the metabolism. For this reason, in vitro, 3D tissue culture, and in vivo models are designed for the preclinical drug evaluation studies, in which the targeted disease defines the metabolites needed to be monitored. To illustrate, the production of the metabolites, such as lactate or pyruvate, can be targeted to assess the efficacy of a drug candidate against cancer cells, as cancer cells have an altered energy metabolism. Glucose, lactate, and glutamine are other energy resources for the cancer cells. Therefore, the consumption of these molecules in addition to the other intracellular metabolites, such as enzyme and cofactors, involved in the energy metabolism were also monitored in several drug discovery studies [97,148,149]. The LOC systems used for the detection of different cytokines released from immune cells [53,54,89,92] can be employed if the tested drugs are associated with a possible immune response [150]. ROS emerge in both cancer promoting and anticancer studies [151,152]. Different LOC systems integrated with optical [40,41,42,45,46,47,153], and electrochemical [83,84,85,86,87] methods for sensing ROS were reported and can be used to assess the oxidative stress and relevant signal transduction mechanisms while determining both the efficacy of a potential drug molecule and its toxicity.

Another area where LOC systems add value is systems biology which aims to understand the structure and dynamics of a system through quantitative extensive molecular and phenotypic characterisations [154,155]. Interdisciplinary work is required to design the experimental flow during which genomics, transcriptomics, proteomics, and metabolomics are used to gather comprehensive understanding of the complex biological systems [154]. The integration of microfluidics with the systems biology gave rise to high-throughput technologies used for molecular and protein analyses at the organism and single cell level [156,157]. Embryonic development has been studied using LOC systems which targeted glucose, lactate, and pyruvate metabolites of mouse and bovine embryos [102,158,159]. There are also other LOC devices used for the morphological characterisations of *Drosophila* [160,161], *Caenorhabditis elegans* [162,163,164], zebra fish (*Danio rerio*) [165], and plant systems such as *Arabidopsis thaliana* [166].

Development of drug/gene/cell therapies require standardised large-scale biomanufacturing processes. Microfluidics provides great advantages due to the ability to synthesise and deliver nanoparticles, drugs, or genes into single cells that will program cells to secrete therapeutic products [167]. LOC devices allowed fabrication of large amounts of nanomedicine with high reproducibility and efficiency [168,169]. Cell therapy involves stem cells and the optimisations for the therapy requires large amount of cells, time, and can be highly labour and economic resources intensive when cultured with conventional techniques. LOC devices have been used to culture single stem cells, to trigger their differentiation due to mimicking physiological microenvironment, and to sort each differentiated cell enabling high-throughput screening capability [170]. The usage of different sensors within these LOC devices increases the versatility of the system. The most common sensing approach is fluorescence-based in which certain cellular compartments, the expression of fluorescent proteins, or the intrinsic fluorescence of a fabricated or delivered nanoparticle can be imaged using fluorescence microscope [167,171].

Bacteria, fungi, virus, and microalgae are microbes which are subject of many research areas due to the potential of them to be pathogenic, be mini factories producing clinically and industrially significant biomolecules such as antibiotics, or be environmentally significant. Nucleic acid metabolites were used to detect and identify the pathogenic microbes, and select the microbes with superior characteristics. These nucleic acids were detected using various LOC devices which perform polymerase chain reaction (PCR) and loop mediated isothermal amplification (LMAP) on a chip with remarkable efficiency, accuracy, sensitivity, and automation [172,173]. Recombinant proteins were used as the biomarkers for the selective profiling of a microbial library [50,58]. In addition, L-tryptophan, lactate, and *p*-coumaric acid were detected as biomarkers for the bioprocess monitoring [49,101,106]. Droplet microfluidics contribute to the emergence of excellent high-throughput screening platforms requiring sub-nL sample volumes that allow single cell manipulation.

## 5. Challenges and Future Perspectives

LOC sensing devices can be used for a wide range of applications when integrated or connected to single and multi- cellular biological systems. Intracellular metabolite detection requires optical probes that are able to penetrate the cell membrane where the cells can be maintained and/or analysed in the LOC device. The limited variety of metabolite probes does not allow the extensive usage of this approach. Moreover, the effect of introducing sensing elements into the individual cells on the normal cellular functions is not fully understood. Therefore, in the current LOC devices, the use of intracellular sensors prevents the post-analysis usage of cells. Similarly, the electrophoresis-based methods require rapturing of cell membrane to reach the intracellular content and so cannot allow the reuse of the analysed cells. In order to address these challenges, label-free single cell and tissue imaging methods are developed with high spatial resolution capable of analyte quantification. Digital holographic imaging, a type of quantitative phase microscopy, is a powerful technique with nanometric resolution allowing the visualisation of cell morphology and quantification of intracellular dynamics due to the naturally occurring phase contrast agent of cells, which is the optical path difference induced by the intracellular components [174]. In the studies where detection of intracellular genetic reporters is required, the conventional fluorescence-based microscopy is not sufficient. Photoacoustic imaging can be used for this purpose and it is a powerful technique that relies on the conversion of optical signal into acoustic waves and has been used to detect and quantify intracellular proteins in addition to reconstruct 3D images of the studied tissues [175]. Another label-free method is Differential Detection Photothermal Interferometry (DDPI) implemented for the quantitation of absorbance based on a phase shift caused by the photothermal effect [176]. DDPI can be used to quantify the metabolites which are naturally absorbing light, such as haemoglobin protein.

The detection of unstable metabolites (such as ROS or RNS) requires the sensors to be spatially very close to the cells. Since adherent cells do not proliferate near those sensors, the distribution of metabolite concentration around them is less than at distant locations, and so this approach fails to give accurate results representing the whole system. For the suspension cell cultures, additional separating elements must be included to prevent interaction of sensors with the cells. Moreover, the reaction products generated after sensing can accumulate and affect the cell behaviour. Complete separation of sensors from the cells will address these issues. Enzymatic electrochemical sensors are extensively used due to their high selectivity and short response time. Poor long-term stability limits their wide range application because enzymes lose their initial activity over the time, and thus the sensor requires calibrations. Non-enzymatic approaches used in electrochemical studies aiming to solve stability issues suffer from providing less selectivity and therefore require further analyses to obtain both stable and highly selective sensors. The exclusion of signal coming from the sample medium itself can help solving this issue. In addition, the formation of a second product obtained after reacting with the target metabolite can also improve the selectivity given that the product has distinctive detectable signal.

Contrary to ROS or RNS, protein metabolites are relatively stable and are detectable by means of both optical and electrochemical methods with affinity-based sensing approach. The irreversible reaction between the sensing probe and the metabolite limits the reusability of the sensors necessary for long-term monitoring. This challenge can be addressed with more stable aptamer-based sensors providing controllable reversibility of analyte binding status. The long incubation times of protein sensors is also not conducive to real-time monitoring. The usage of microcapillary electrophoresis can decrease these long incubation times.

Addressing the aforementioned issues will allow the multiplex real-time analysis of cell metabolites providing novel insights into the cellular functions. Miniaturised LOC devices can be used as a platform to culture an individual cell, an organoid, and even whole organs. Integration of sensing compartments will dramatically increase the knowledge acquired from these systems utilising sub-nL sample volumes and providing real-time continuous monitoring. Discovery of new biomarkers and having complete understanding of the biological role of these biomarkers will lead to the development of new sensing approaches as well as the development of new treatment strategies. The use of open-source hardware can significantly decrease the manufacturing costs providing a versatile platform compatible with various sensors. The more cost-efficient device fabrication technologies will help to develop commercial, easier accessible, and affordable LOC cell culture monitoring devices. Implementing different types of microfluidics such as digital microfluidics will maximise the control over the sample manipulation. Overall, LOC devices promise great improvements in conventional lab-scale metabolite monitoring protocols. The state-of-the-art platforms need to be fully optimised and become available for a wider community to transform how biology is studied.

## Figures and Tables

**Table 1:** LOC systems used for optical metabolite detection.

Metabolite	Method	DL	Linear Range	Cell Type	Stimulant	Location	Purpose	FC	Ref.
H_2_O_2_	Fluor.	5.6 nM	0.02–5 µM	Macrophage	PMA	Intra.	Oxidative stress	Electrophoresis	[40]
H_2_O_2_	Fluor.	90 nM	0.0072–1 µM	Hepatocyte	No	Intra.	Single cell analysis	Cell lysis Electrophoresis	[41]
H_2_O_2_	Fluor.	0.1 nM	0.055–11.6 amol	Hepatocyte	Ethanol	Intra.	Single cell analysis	Electrophoresis	[42]
H_2_O_2_	Colour.	40 nM	NR	Microalgae	Cd^2+^, QD	Extra.	Ecotoxicology	Valve Microsieve Continuous sensing	[43]
H_2_O_2_	Fluor.	NR	NR	Glyoblastoma HUVEC	α-lipoic acid catechin ascorbic acid	Intra.	Antioxidant screening	Cell co-culture Tumour microvascular structure	[44]
O_2_*^−^	Fluor.	10 nM	0.08–5 µM	Macrophage	PMA	Intra.	Oxidative stress	Electrophoresis	[40]
O_2_*^−^	Fluor.	4.8 nM	0.01–2 µM	Rat adrenal medulla	No	Intra.	Single cell analysis	Cell lysis Electrophoresis	[45]
NO	Fluor.	5.3 nM	0.0075–5 µM	Rat adrenal medulla	No	Intra.	Single cell analysis	Cell lysis Electrophoresis	[45]
ROS	Fluor.	6.9 amol	NR	Erythrocyte	H_2_O_2_	Intra.	Oxidative stress	Cell lysis Electrophoresis	[46]
ROS	Fluor.	NR	NR	Endothelial	Glucose Shear stress	Intra.	Oxidative stress	Cell culture	[47]
ROS	Fluor.	NR	NR	Fibroblast adenocarcinoma spheroid	Nano-TPP	Intra.	PDT analysis	Cell-co culture Spheroid formation	[48]
Cysteine	Fluor.	0.02 µM	60.5–7260 amol	Hepatocyte	Ethanol	Intra.	Single cell analysis	Electrophoresis	[42]
Glutathione	Fluor.	0.01 µM	38.5–17600 amol	Hepatocyte	Ethanol	Intra.	Single cell analysis	Electrophoresis	[42]
Glutathione	Fluor.	NR	NR	Glyoblastoma HUVEC	α-lipoic acid catechin ascorbic acid	Intra.	Antioxidant screening	Cell co-culture Tumour microvascular structure	[44]
*Glutathione*	*Fluor.*	*0.5 amol*	*NR*	*Erythrocyte*	*H_2_O_2_*	*Intra.*	*Oxidative stress*	*Cell lysis* *Electrophoresis*	*[46]*
L-tryptophan	Fluor.	NR	<1 g/L	*E. coli*	No	Intra.	Synthetic biology	Valve Droplet formation High throughput screening	[49]
Tyrosine	Fluor.	NR	NR	*S. cerevisiae*	No	Extra.	Protein engineering Synthetic biology	Droplet formation High throughput screening	[50]
Urea	Colour.	2 µM	0–1 mM	Hepatocyte	No	Intra.	Hepatocyte culture monitoring	Mixer Waveguide	[51]
ATP	Biol.	0.2 µM	0.2–50 µM	*E. coli*	No	Intra.	Single cell analysis	Electrophoresis	[52]
IL-2	Fluor.	NR	0–400 ng/mL	*T lymphocyte*	PMA, Ionomycin	Extra.	Immunology	Cell capture	[53]
IL-6	Fluor.	143 pg/mL	NR	Monocyte	LPS, LTA, CpG-B, Flagellin, poly I:C	Extra.	Immunology	Cell capture Cell culture Reconfigurable barriers	[54]
IL-10	Fluor.	177 pg/mL	NR	Monocyte	LPS, LTA, CpG-B, Flagellin, poly I:C	Extra.	Immunology	Cell capture Cell culture Reconfigurable barriers	[54]
TNF-α	Fluor.	109 pg/mL	NR	Monocyte	LPS, LTA, CpG-B, Flagellin, poly I:C	Extra.	Immunology	Cell capture Cell culture Reconfigurable barriers	[54]
IFN-γ	Fluor.	NR	0–100 ng/mL	T lymphocyte	PMA, Ionomycin	Extra.	Immunology	Cell capture	[53]
TGF-β1	Fluor.	21 pM	0–300 pM	Hepatocyte	No	Extra.	GF secretion monitoring	Cell culture Hydrogel barrier	[55]
*HGF*	*Fluor.*	*6 pM*	*0–40 pM*	*Hepatocyte*	*No*	*Extra.*	*GF secretion monitoring*	*Cell culture* *Hydrogel barrier*	*[55]*
VEGF_165_	Lum.	0.17 pM	0.52–52 pM	*Epidermoid carcinoma*	Paclitaxel	Extra.	Protein-DNA interaction	Hydrodynamic focusing	[56]
Bile acid	Colour.	2.1 µM	0–150 µM	Hepatocyte spheroid	Ethanol	Extra.	Toxicology	Droplet formation Spheroid culture	[57]
Streptavidin	Fluor.	NR	1–40 mg/L	*S. cerevisiae*	No	Extra.	Protein engineering Synthetic biology	Droplet formation High throughput screening	[50]
Lactate dehydrogenase	Fluor.	0.5 U/L	0–80 U/L	Hepatocyte spheroid	Ethanol	Extra.	Toxicology	Droplet formation Spheroid culture	[57]
Recombinant enzymes	Fluor.	NR	NR	*Yarrowia* *lipolytic*	No	Extra.	Protein engineering Library screening	Droplet formation Yeast culture High throughput screening	[58]
Metalloproteinase 9	Fluor.	2.3 nM	0–80 nM	Lymphoma	PMA	Extra.	Single cell analysis	Cell capture Hydrogel islands Reconfigurable barriers	[59]
B[a]P	Electro-chemilum.	NR	NR	N/A DNA oligonucleotide	No	N/A	Genotoxicity	High throughput screening	[60]

CFC: functional components, Intra: intracellular, Extra: extracellular, DL: limit of detection, ROS: reactive oxygen species, O_2_*^−^: superoxide radical, H_2_O_2_: hydrogen peroxide, NO: nitric oxide, IL-2: interleukin 2, IL-6: interleukin 6, IL-10: interleukin 10, TNF-α: tumour necrosis factor –α, IFN-γ: interferon –γ, TGF-β1: transforming growth factor –β1, HGF: hepatocyte growth factor, ATP: adenosine triphosphate, HGF: hepatocyte growth factor. B[a]P: benzo[a]pyrene-7,8-dihydrodiol-9,10-epoxide. HUVEC: human umbilical vein endothelial cells, nano-TPP: meso-tetraphenylporphyrin, NR: not reported, N/A: not applicable, Colour: colourimetric-based, Fluor: fluorescence-based, Electrochemilum: electrochemiluminescence-based, Lum: luminescence-based, Biolum: bioluminescence-based, QD: quantum dot.

**Table 2:** LOC systems used for the electrochemical metabolite detection. Lay out like Tab 1

Metabolite	Method	DL	Linear Range	Cell Type	Stimulant	Purpose	Functionality	Ref
H_2_O_2_	CV, SWV	NR	1–800 µM	Rat heart tissue	No	Heart pathophysiology	Tissue culture Electrical stimulation	[83]
H_2_O_2_	Amp.	0.2 µM	0–100 µM	Hepatocyte	Ethanol	Toxicology	Cell culture	[84]
H_2_O_2_	Amp.	NR	NR	Macrophage	Calcium ionophore	Oxidative stress	Cell culture	[85]
O_2_*^−^	Amp.	38 nM	0.75–3.5 µM	Breast carcinoma	PMA	Oxidative stress	Integrated into culture flask	[86]
NO*	Amp.	NR	NR	Macrophage	Calcium ionophore	Oxidative stress	Cell culture	[85]
NO_2_^−^	Amp.	NR	NR	Macrophage	Calcium ionophore	Oxidative stress	Cell culture	[85]
ONOO^−^	Amp.	NR	NR	Macrophage	Calcium ionophore	Oxidative stress	Cell culture	[85]
Total ROS and RNS	Amp.	NR	NR	Macrophage	Calcium ionophore	Oxidative stress	Cell culture Continuous sensing	[87]
TNF-α	SWV	5 ng/mL	5–100 ng/mL	Monocyte	PMA, Ionomycin	Intercellular communication	Cell culture Reconfigurable barrier formation Analyte concentration	[88]
TNF-α	SWV	5.46 ng/mL	9–88 ng/mL	T lymphocyte Monocyte	PMA, Ionomycin	Cell secretion	Cell capture Multiplex sensing	[89]
GST-α	EIS	0.01 ng/mL	0.1–100 ng/mL	Hepatocyte Cardiomyocyte	Acetaminophen, Doxorubicin	Drug screening	Valve Automated Multi-functional sensing platform	[90]
TGF-β1	SWV	1 ng/mL	0–250 ng/mL	Hepatic stellate	PDGF	Fibrosis	Cell culture Reconfigurable barrier formation	[91]
IFN-γ	SWV	5 ng/mL	5–100 ng/mL	T lymphocyte	PMA, Ionomycin	Cell secretion	Cell capture Reconfigurable microcup formation	[92]
IFN-γ	SWV	6.35 ng/mL	9–130 ng/mL	T lymphocyte Monocyte	PMA, Ionomycin	Cell secretion	Cell capture Multiplex sensing	[89]
Transferrin	Amp.	0.03 ng/mL	10–4000 ng/mL	Hepatocyte	Acetaminophen	Toxicology	Valve Automated Multi-functional sensing platform	[93]
Albumin	Amp.	0.03 ng/mL	15–4000 ng/mL	Hepatocyte	Acetaminophen	Toxicology	Valve Automated Multi-functional sensing platform	[93]
Albumin	EIS	0.09 ng/mL	0.1–100 ng/mL	Hepatocyte Cardiomyocyte	Acetaminophen, Doxorubicin	Drug screening	Valve Automated Multi-functional sensing platform	[90]
CK-MB	EIS	0.0024 ng/mL	0.01–10 ng/mL	Hepatocyte Cardiomyocyte	Acetaminophen, Doxorubicin	Drug screening	Valve Automated Multi-functional sensing platform	[90]
Lactate	Amp.	65 fmol	65–266 fmol	Cardiac myocyte	FCCP, Saponin	Single cell analysis	Cell culture Working volume of pL	[94]
Lactate	Amp.	7.4 µM	0–101.5 µM	Rabbit myocyte	Electric stimulation	Single cell analysis	Cell culture Working volume of pL	[95]
Lactate	Amp.	0.16 mM	0.2–10 mM	Rat cardiomyocyte	FCCP	Energy metabolism	Continuous monitoring	[96]
Lactate	Amp.	90 µM	0–3 mM	Brain cancer	*Cytochalasin B*	Drug screening	Cell culture Continuous real-time multiparameter monitoring	[97]
Lactate	Amp.	NR	0.5–10 mM	Hepatocyte	*Rotenone*	Toxicology	Continuous monitoring	[98]
Lactate	Amp.	NR	0.06–0.3 mM	Colorectal adenocarcinoma	*Triton-X100*, *CuCl_2_*, *Acetaminophen*	Toxicology	Enzyme µ-bioreactor	[99]
Lactate	Amp.	7 µM	0–1 mM	Colon carcinoma spheroid	No	Energy metabolism	Hanging drop Spheroid culture	[100]
Lactate	Amp.	NR	0–900 mM	*Saccharomyces cerevisiae, Lactobacillus acidophilus*	No	Bioprocess monitoring	Multiplex real-time sensing	[101]
Lactate	Amp.	0.4 µM	0–6 mM	Bovine embryo	No	Energy metabolism	Embryo culture Multiplex real-time sensing	[102]
Norepinephrine	CV	NR	0–400 µM	Chromaffin	Tyrode’s solution with 50 mM K^+^	Exocytotic transmitter release profiling	Real-time sensing	[103]
Norepinephrine	CV	NR	10–500 µM	Chromaffin	Carbachol, KCl, PACAP	Exocytotic transmitter release profiling	Cell trap Real-time sensing	[104]
Epinephrine	CV	NR	10–500 µM	Chromaffin	Carbachol, KCl, PACAP	Exocytotic transmitter release profiling	Cell trap Real-time sensing	[104]
Dopamine	CV	NR	10–500 µM	Chromaffin	Carbachol, KCl, PACAP	Exocytotic transmitter release profiling	Cell trap Real-time sensing	[104]
PCA, 5-MCA, PYO	SWV	NR	NR	*P. aeruginosa*	No	Metabolite profiling in biofilm	Cell culture High-throughput screening	[105]
p-coumaric acid	SWV	NR	0.125–2 mM	*E. coli*	Tyrosine	Bioprocess monitoring	Filtration	[106]
β-gal	Amp.	NR	NR	*S. cerevisiae*	17β-estradiol, Tamoxifen, Vitamin K3	Hormone active chemical screening	Electrophoresis Single cell analysis	[107]

FC: functional component, DL: detection limit, ROS: reactive oxygen species, RNS: reactive nitrogen species, H_2_O_2_: hydrogen peroxide, O_2_*^−^: superoxide radical, ONOO^−^: peroxinitrite, NO*: nitric oxide radical, NO_2_^−^: nitrogen dioxide, TNF-α: tumour necrosis factor -α, GST-α: glutathione S-transferase α, IFN-γ: interferon γ, TGF-β1: transforming growth factor -β1, FCCP: carbonyl cyanide-4-phenylhydrazone, PCA: phenazine-1-carboxylic acid, 5-MCA: 5-methyl-phenazine-1-carboxylic acid, PYO: pyocyanin, PMA: phorbol 12-myristate 13-acetate, PACAP: pituitary adenylate cyclase activating polypeptide, SWV: square wave voltammetry, Amp: amperometric, CK-MB: creatine kinase MB, DNP-BSA: dinitrophenylated bovine serum albumin, β-gal: β galactosidase.
